# Population Genetics of the São Tomé Caecilian (Gymnophiona: Dermophiidae: *Schistometopum thomense*) Reveals Strong Geographic Structuring

**DOI:** 10.1371/journal.pone.0104628

**Published:** 2014-08-29

**Authors:** Ricka E. Stoelting, G. John Measey, Robert C. Drewes

**Affiliations:** 1 Department of Herpetology, California Academy of Sciences, San Francisco, California, United States of America; 2 Department of Forest and Wildlife Ecology, University of Wisconsin, Madison, Wisconsin, United States of America; 3 Centre for Invasion Biology, Department of Botany and Zoology, University of Stellenbosch, Stellenbosch, South Africa; University of Arkansas, United States of America

## Abstract

Islands provide exciting opportunities for exploring ecological and evolutionary mechanisms. The oceanic island of São Tomé in the Gulf of Guinea exhibits high diversity of fauna including the endemic caecilian amphibian, *Schistometopum thomense*. Variation in pigmentation, morphology and size of this taxon over its c. 45 km island range is extreme, motivating a number of taxonomic, ecological, and evolutionary hypotheses to explain the observed diversity. We conducted a population genetic study of *S. thomense* using partial sequences of two mitochondrial DNA genes (ND4 and 16S), together with morphological examination, to address competing hypotheses of taxonomic or clinal variation. Using Bayesian phylogenetic analysis and Spatial Analysis of Molecular Variance, we found evidence of four geographic clades, whose range and approximated age (c. 253 Kya – 27 Kya) are consistent with the spread and age of recent volcanic flows. These clades explained 90% of variation in ND4 (*φ_CT_* = 0.892), and diverged by 4.3% minimum pairwise distance at the deepest node. Most notably, using Mismatch Distributions and Mantel Tests, we identified a zone of population admixture that dissected the island. In the northern clade, we found evidence of recent population expansion (Fu's *F_s_* = −13.08 and Tajima's *D* = −1.80) and limited dispersal (Mantel correlation coefficient = 0.36, *p* = 0.01). Color assignment to clades was not absolute. Paired with multinomial regression of chromatic data, our analyses suggested that the genetic groups and a latitudinal gradient together describe variation in color of *S. thomense*. We propose that volcanism and limited dispersal ability are the likely proximal causes of the observed genetic structure. This is the first population genetic study of any caecilian and demonstrates that these animals have deep genetic divisions over very small areas in accordance with previous speculations of low dispersal abilities.

## Introduction

Islands are self-contained natural laboratories and provide the backdrop to many of Darwin's and Wallace's observations that culminated in the theory of evolution [1]. Continuing in this role, they provide contemporary biologists with the opportunity to investigate ecological and evolutionary mechanisms [2]–[8]. On truly oceanic islands, isolation and competition for open ecological niches promote speciation [6], placing these locales in a prominent role when considering conservation of biodiversity [9]. In this context, the use of molecular phylogenetics has elucidated insular taxonomic relationships, providing insight into the mechanisms of island colonization and ecological radiations [10], [11]. Many studies looking at insular species have concentrated on the examination of taxonomic relationships between islands [12]–[14], but, with their wealth of endemics, islands also provide an opportunity to study intraspecific population-level relationships in great detail.

Notable for its high endemism in both flora and fauna – including approximately 80 plants [15], 17–18 butterflies [16], 17 birds [17], 14 reptiles and five amphibians (RCD *pers. obs*. 1 Aug 2012) – São Tomé is one of three oceanic islands in the Gulf of Guinea that rose along the Cameroon Line roughly 13 Mya [18]–[19], and the largest at 836 km^2^, with the highest peak at 2024 m. Over the course of its existence, volcanic activity has reshaped topography [20]–[21] with recent data showing that it was active throughout the Pleistocene [19], likely contributing to the diversity observed; most recent flows have been placed between 0.85 and 0.03 Mya [19]. In addition, multiple independent colonization events have contributed to diversity, evidenced by phylogenetic relationships of geckos [22]–[23], skinks [24], and multiple snake species [25] on São Tomé and nearby islands.

The presence of salt-intolerant taxa on truly oceanic islands has garnered particular interest because mechanisms accounting for presence imply dispersal across marine environments, but direct dispersal is assumed to be limited by physiology. However, recent phylogenetic studies have suggested the existence of delivery mechanisms, in particular oceanic currents, which carry flotsam [26]–[29], as well as the importance of decreased salinity of surface waters [27]. Such studies imply that colonization may be more common than previously assumed [30]. Here, we focus on an insular taxon of the sparsely-documented amphibian Order Gymnophiona – *a.k.a.*, caecilians – for which very little intraspecific information exists [31].

**Figure 1:**
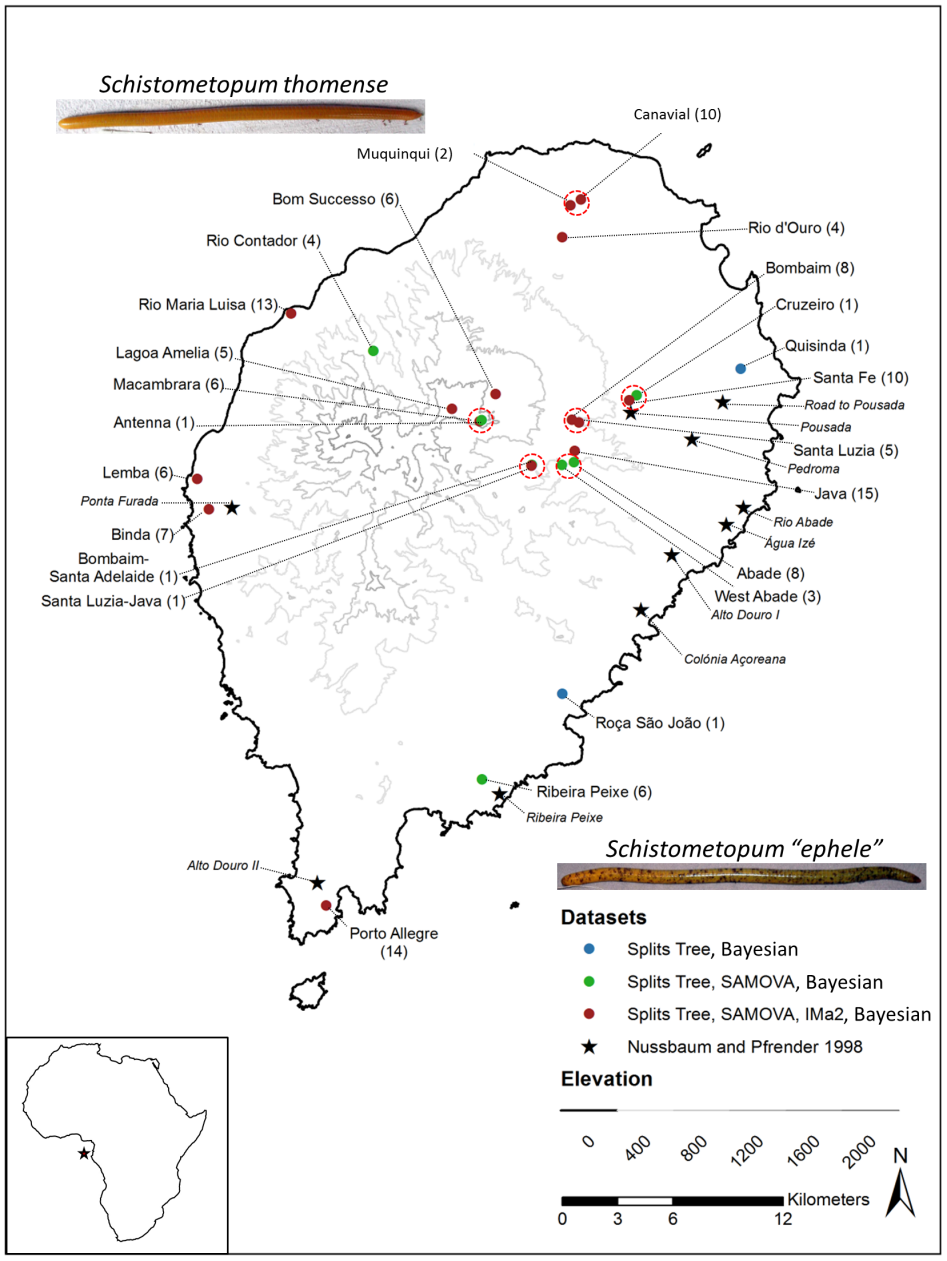
*Schistometopum thomense* collection localities, São Tomé, Republic of São Tomé and Príncipe. Numbers in parentheses indicate number of specimens available for genetic analyses. Legend indicates specimens used in Splits Tree, Bayesian, SAMOVA, and/or IMa2 analyses, or discussed by Nussbaum and Pfrender [36] in morphological comparisons. Dashed, red ovals indicate populations lumped for SAMOVA analyses. Photographs show examples of clear and flecked morphs as *Schistometopum thomense* and *Schistometopum* “*ephele*”, respectively. Red star in lower left panel indicates relative position of São Tomé to continental Africa.

The caecilian amphibian *Schistometopum thomense* (Barboza du Bocage) [32] is a biogeographical enigma as it is endemic to the oceanic island of São Tomé, 225 km off the western coast of continental Africa, while its lone congener, *Schistometopum gregorii*, occurs on the far eastern coast of continental Africa (Kenya and Tanzania) [33]. Until recently, three endemic caecilian species were recognized from São Tomé, their differences based principally on coloration and head morphology. Presence of purple-brown flecking and a more pointed head shape differentiated *Schistometopum ephele* Taylor [34] from *S. thomense* (photographs, Figure 1). And, *Schistometopum brevirostre* (Peters) [35] was distinguished by Taylor [34] for its pale bluish coloration and slightly differentiated morphometric characters. But, subsequent morphological analyses suggested this division to be erroneous [36]; Nussbaum and Pfrender [36] synonymized all species to *S. thomense*, recognizing that criteria used to designate *S. ephele* Taylor were expressions of sexual dimorphism and of possible clinal variation from north to south on the island, and confirming that the third taxon – *S. brevirostre* – had been described without knowledge of the initial description [36]–[37]. Nevertheless, Nussbaum and Pfrender [36] found the range in color variation across this small island to be extreme and suggested limited dispersal ability for these caecilians as an explanation. The hypothesis that *S. thomense* may have limited dispersal ability receives further support from the finding that this species exhibits Bergmann's rule; Measey and Van Dongen [38] found that body mass doubled and length increased by nearly 50% along a 1,000 m elevational transect. Because subterranean organisms usually do not broadcast their biological parameters through morphology or easily-observable ecological niches, many questions remain about their taxonomy and ecology. For *S. thomense*, examining genetic variation at the species level offers indirect means to understand microevolutionary processes acting within this fossorial species.

In order to assess population structuring of *S. thomense*, we investigated the distribution of genetic diversity using mitochondrial DNA (mtDNA). In particular, we were interested in whether previously proposed taxonomic (i.e., multiple lineages) or clinal variation (i.e., one lineage) hypotheses could better explain the high phenotypic variation exhibited by caecilians on São Tomé [34], [36], [38]. In addition, we approximated the time to most recent common ancestor (MRCA) of lineages, thereby allowing investigation of the possibility that vicariant events contributed to the apparent diversity among caecilians on the island. As noted by Seyahooei et al. [39], maternally-inherited mitochondrial markers have the capacity to reflect female migration patterns that may be obscured in nuclear DNA (nDNA), which is subject to recombination. Thus, if distinct lineages led to the diversity of caecilians observed on São Tomé, we would expect to see a statistically-supported substructure of population groupings across the island. However, if clinal variation led to this diversity, we would expect subtler differentiation, with genetic distances between populations more closely mirroring geographic distance on São Tomé. If vicariant events had influenced intra-specific structure, we might expect that dates to MRCA and genetic structure correlate with estimated dates and breadth of volcanic activity and sea level change – the only vicariant events for which we have data – on the island.

## Methods

### Ethics statement

Permission to collect, sample and export *Schistometopum thomense* on the island of São Tomé was was granted by Hon. Arlindo Carvalho, Director General, Ministry of Environment, Republic of São Tomé and Príncipe. Ethics clearance for this study was granted by the Office for the Protection of Human and Animal Subjects, San Francisco State University (Protocol # A3-014).

### Study species

*Schistometopum thomense* (Barboza du Bocage) is an elongate, limbless, burrowing amphibian of the Order Gymnophiona (Dermophiidae, formerly Caeciliidae [40]), generally regarded as endemic to the island of São Tomé, though also represented by one specimen purportedly collected in the Ruwenzori region of the Democratic Republic of the Congo [36]. Adults range in total length from roughly 130 to 375 mm [36], [38] and exhibit viviparous reproduction that is not tied to water [36]. These caecilians are one of only a few species that have been bred in captivity and, therefore, have a known age of maturity: 2 years [41]. This caecilian has received disproportionate attention amongst gymnophionans due to its striking yellow coloration and the relative ease with which it can be found, frequenting a variety of habitats from sea level to 1440 m in elevation [36], [38], [41]–[47]. Unflecked morphs appear more common in the north of the island and heavily flecked morphs appear more common in the south of the island, although geographic assignment of morphotype is not absolute [36], [41]–[44], [47]. We found these caecilians to be locally abundant [48], but impacts of land-use change [49] and recent entry into the pet-trade [50] on range and density are unknown.

### Sample collection

In order to assess genetic and chromatic variation within the range of *Schistometopum thomense*, we took photographs of and collected liver tissue from 138 specimens representing 24 geographic locales (“populations”) on the island of São Tomé (Figure 1). Specimens were deposited in the collections of the California Academy of Sciences' Department of Herpetology (CAS), the British Museum of Natural History (BMNH) and of author GJM (Table S1). Specimen collection dates encompass 2000 to 2006. Collection localities span the island, but reflect accessible habitat rather than the complete distribution of these animals. Liver tissue of *Schistometopum gregorii* (MW 03231) was used as an outgroup in phylogenetic analyses.

### Molecular data

Whole genomic DNA was extracted from liver tissue, using either phenol-chloroform [51] or DNeasy (Qiagen Inc., Valencia, CA) extraction protocols. From these samples, approximately 900 base pairs of NADH dehydrogenase subunit 4 (ND4) and associated tRNAs (histidine, serine and partial leucine) were amplified using the primers ND4 and Leu [52] in 100 µL and 10 µL reactions by hot-start PCR [51] and non-hot-start methods, respectively. Hot-start reactions were conducted in 100 µL volumes (25 µL Top Mix: 2× buffer, 0.8 mM dNTPs, 1 µM each primer; 75 µL Bottom Mix: 0.67× buffer, 2 mM MgCl_2_, 0.033 U Bioline Taq, 10 µL template DNA), and used the temperature profile: 94°C for 7 min., 40 cycles of 94°C for 30 sec., 46°C–50°C for 30 sec., 72°C for 1 min., followed by 72°C for 7 min. Non-hot-start reactions were conducted in 10 µL volumes (1× buffer, 0.175 mM dNTPs, 1.25 mM MgCl_2_, 0.05 U Bioline Taq, 0.54 µM of each primer, 2 µL template DNA) and used the temperature profile: 94°C for 7 min., 25 cycles of 94°C for 30 sec., 50°C for 30 sec.-1 min., 72°C for 1 min., followed by 72°C for 7 min. In addition to ND4, approximately 600 base pairs of the small ribosomal subunit 16S were amplified using the primers 16sar and 16sbr [53] in 10 µL non-hot-start reactions similar to those described above, substituting Apex Taq for Bioline Taq, with the following temperature profile: 94°C for 3 min., 39 cycles of 94°C for 30 sec., 50°C for 30 sec., 72°C for 1 min., followed by 72°C for 5 min., and dropping to 25°C for 2 minutes.

PCR product was cleaned using the Promega Wizard PCR Preps DNA Purification System (Promega, Madison, WI) following manufacturer's protocol, or with ExoSAP-IT (USB Corporation, Cleveland, OH), using the following reaction conditions: 7.5 *μ*L volumes (0.83 *μ*L 10×SAP Buffer, 0.15 *μ*L SAP Enzyme and 0.08 *μ*L Exo I Enzyme per sample), incubating at 37°C for 15 min., 80°C for 15 min., then cooling to 4°C. Based on visual strength of bands in an agarose gel, 0.5–1.0 *μ*L Wizard Prep product or 1.0–4.5 *μ*L of ExoSAP-IT reaction product were then used in cycle sequencing reactions.

Cycle Sequencing was performed on either a Perkin Elmer 9600, Perkin Elmer 9700 or BioRad thermocycler, following ABI prism instructions for either Big Dye version 2.0 (50 cycles of 96°C for 10 sec, 45°C for 5 sec, and 60°C for 4 min) or Big Dye version 3.1 (either 50 cycles of 96°C for 10 sec, 50°C for 5 sec, and 60°C for 4 min, or 25 cycles of 96°C for 15 sec, 50°C for 15 sec, and 60°C for 4 min) (Perkin-Elmer, Norwalk, CT), depending on which reagent was used. For all samples, amplified products were sequenced in both the 3′ and 5′ directions, using the original PCR primers. Nucleotide order was read by an ABI-PRISM 3100 Genetic Analyzer (Applied Biosystems, Norwalk, CT). Base calls and editing were resolved by eye using Sequencher versions 3.1 and 4.2 (Gene Codes Corporation, Ann Arbor, MI). Confirmation of ND4 as the amplified product was made with BLAST searches (http://www.ncbi.nlm.nih.gov/BLAST/) in GenBank [54].

To confirm that evolutionary patterns in amplified sequences were not obscured by strong selective pressure, standard neutrality tests – Tajima's *D* [55]–[57], Ewens-Watterson homozygosity [58]–[59], Slatkin's exact test based on Ewens' sampling [60]–[61], Fu's *F*_s_ [62] and Chakraborty's test of population amalgamation [63] - were run in Arlequin v 3.5 [64].

### Clinal or taxonomic substructure?

We conducted Bayesian phylogenetic analysis in MrBayes version 3.2.0 [65]. The initial model framework for this search was determined with MrModelTest 2.2 [66] using AIC. A 50% majority–rule consensus tree was then generated in MrBayes, using 4 MCMC chains that ran for 120,000 generations, sampling every 10 generations, and using burn-in of 20% via the CIPRES Science Gateway [67]. We assessed stationarity of the model by looking at whether potential scale reduction factor (PSRF) of parameter estimates was close to 1.0 and whether the log probability of the data across MCMC generations was free of any particular pattern.

Geographic substructure was examined in the ND4 dataset using spatial analysis of molecular variance (SAMOVA [68]). This analysis attempts to define the most likely number and composition of geographically-differentiable genetic groups within a dataset by considering the frequency of haplotypes, and the pairwise genetic differences between haplotypes, within and among k groups relative to that in a total dataset (*φ*_CT_). Because SAMOVA performs poorly with datasets populated by low sample-size locations, disjunct sampling localities represented by one individual only were excluded and neighboring and/or remaining low sample size locales (*n*≤2) were grouped with proximate larger populations, creating a dataset of 136 individuals from 16 sample sites (SAMOVA sample sites represented in green and blue and lumped populations circled by dashed red lines in Figure 1). Configurations of two through ten possible groupings (k) were tested. Best resulting groups were then characterized by amount of haplotype diversity, *h* [69], and nucleotide diversity, *π* [70], and subjected to neutrality tests (Fu's *F_s_* and Tajima's *D*) to determine whether any group might be out of mutation drift equilibrium [55]–[57], [62], [71]; these tests were run in Arlequin. Fu's *F*_S_ and Tajima's *D* tests are sensitive to frequencies of alleles within a sample and will generate significantly negative values when an excess of rare frequency alleles exists, a condition that is expected after recent population expansion. In the instance that a group returned significant values of either of these tests, mismatch distribution analyses were run [72]–[73] to confirm and to estimate the timing of population expansion. In order to do this, a model of demographic expansion was assumed, *t* = *τ*/(2*u*), in which *t* is generation time, *τ* is the age of the expansion in mutational units (estimated in the model of demographic expansion) and *u* is the sum of the per nucleotide mutation rate for the region sequenced [72]–[73]. For *S. thomense*, generation time was set at two years based on reported ages at first reproduction for captive females [41] and *u* was estimated from mutation rates of ND4 in amphibians that were available in the literature (the mean of estimates in Arntzen et al. [74] and Wielstra et al. [75]).

To further examine relationships among groups, the full ND4 dataset (138 individuals from 24 populations; sample sites represented by all colored dots in Figure 1) was modeled using a split Neighbor-Net algorithm [76]–[77] in SplitsTree v 4.11.3 [78]. In addition, uncorrected pairwise genetic distances were calculated using Arlequin. Evidence for isolation-by-distance was explored with Mantel tests [79] in Arlequin, assessing the correlation of Euclidean distances between sample locales with pairwise-*F*_ST_ values based on uncorrected pairwise distances between haplotypes at those sites; these tests were run for the island sample as a whole and separately for SAMOVA-derived groups that contained *n*>2 populations (number of permutations = 16,000).

### Divergence times

To estimate population divergence times, we used SAMOVA-identified population groupings in a coalescent approach, with an Isolation with Migration approach using software IMa2 v 2.0 [80]. The IMa2 software uses a Felsenstein framework to run Markov chain Monte Carlo (MCMC) simulations permitting likelihood-based analyses [81] and allows estimation of time to most recent common ancestor for related phylogenetic groups. We used our Bayesian tree (see above) as a prior input topology for the sampled populations. MCMC parameterization of the model included a burn-in duration of 1,000,000 steps, 10,000 genealogies saved, and geometric heating with 25 chains (0.95 as first and 0.9 as second chain heating parameters). Because preliminary runs showed very different results for populations from the North versus southerly populations, we used a prior file to allow different priors for different populations. Priors were determined by using shorter analysis trials. Three final duplicate runs were submitted to the remote computer cluster running the program IMa2 at Cornell University via internet upload (http:\\cbsuapps.tc.cornell.edu\IMa.aspx). Each run of IMa2 returned a model parameter *t* for each lineage pair, as well as the marginal posterior probability densities with 95% upper and lower limits [80]. To estimate divergence time in years (*T*), the geometric mean of the mutation rate (*U*) for the markers sequenced was used: *T* = *t*/*U* [70]–[71]. Mutation rates for each marker (*μ*) per nucleotide site per million years were estimated from amphibian rates for ND4 and 16S in the literature (16S: Vences et al. 2005 [82], ND4: the mean of Arntzen et al. [74] and Wielstra et al. [75]).

### Color

We followed Nussbaum and Pfrender [36] in their scoring of *S. thomense* to one of four different dorsal color classes: 1) light yellow, no brown flecks, 2) dark yellow, no brown flecks, 3) dark yellow, light to moderate brown flecking, 4) dark yellow, heavy brown flecking. Colors were scored by two of us (RES and GJM) from live animals or from images of animals (after anesthesia). Any disagreements in scores were resolved by reviewing images of specimens concerned.

To test whether color variation was best explained by genetic lineages and/or clinal variation, we treated color coding of ND4-sequenced *S. thomense* (*n* = 138) as a nominal response variable (category 4 serving as the reference) in a multinomial logistic model implemented in PROC GLIMMIX of SAS v 9.2 [83]. (We initially attempted to model color as an ordered response variable, but found that the dataset did not meet the assumptions of ordinal logistic regression.) Explanatory variables were latitude (continuous fixed effect), altitude (continuous fixed effect), geology (categorical fixed effect with four levels: alluvium and flood deposits; basaltic lavas 3–8 Mya; basaltic lavas <1 Mya; and pyroclastic/lava cones <0.4 Mya, based on the underlying geological layer presented in Caldeira et al. [21]), and genetic populations (from SAMOVA results, categorical fixed effect with four levels: Central+East, West, North, and South). We selected these explanatory variables based on previous hypotheses or from proxies when data was unavailable. Specifically, latitude was postulated by Nussbaum and Pfrender [36], altitude was found to be important in clinal variation in size [38], geology was selected as a proxy for soil type and vegetation, which are expected to influence caecilian color crypsis [84], and genetic groupings are expected to reveal any taxonomic explanation [34]. Our global model included all main effects described above. To test hypotheses about fixed effects, we ran the global and all nested models, subsequently ranking each model based on AIC_c_ scores [85]. We reported the 95% weight candidate model set and examined the support for various covariates by assessing 95% confidence limits around regression parameters.

## Results

### Sequence information

We sequenced approximately 847 base pairs of mitochondrial ND4 DNA (including flanking tRNAs His, Ser, Leu2 [CUN codon]) and 538 additional base pairs of mitochondrial 16S rRNA from 138 and 26 individual *Schistometopum thomense*, respectively, collected at 24 sample locations across the island of São Tomé (Figure 1, Table S1, GenBank accession numbers KM210341-KM210478 [ND4] and KM210479-KM210488, KM210490-KM210506 [16S]; these sequences aligned with positions 10,829–11,675 [5′-3′, ND4] and 1,939–2,742 [5′-3′, 16S] in the complete mitochondrial genome of *S. thomense* published under GenBank accession GQ244476.1 [86]. Both datasets contained high levels of selectively-neutral heterogeneity: 57 haplotypes and 118 polymorphic nucleotide sites in ND4 and 6 haplotypes and 11 polymorphic nucleotide sites in 16S. These same regions were sequenced in one *Schistometopum gregorii*, collected from Bagamo, Tanzania, (Table S1, GenBank accession numbers KM210507 [ND4] and KM210489 [16s]) for use as an outgroup in phylogenetic analyses.

### Relationships of genetic groups on the island

**Figure 2:**
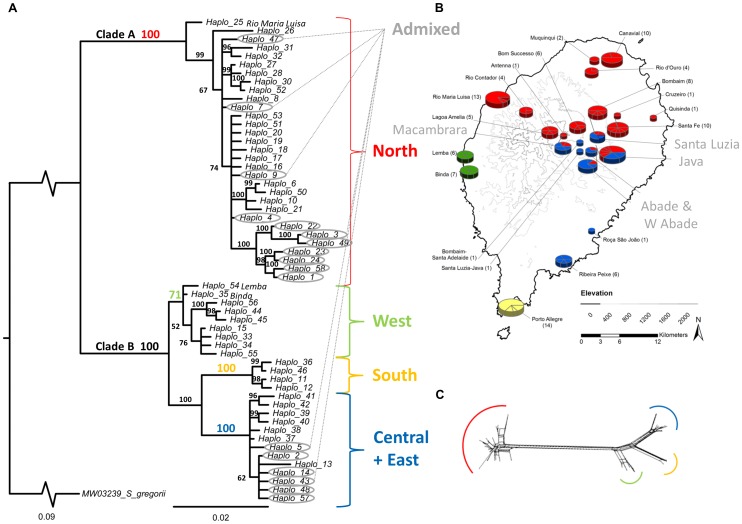
Genetic and geographic relationships of ND4 haplotypes. **A**) **Bayesian Phylogram** (K80+G, 50% Majority-Rule Consensus Tree), with Bayesian Posterior Probabilities (PP) indicated above branches: Color-coding depicts groups “Admixed”, “North”, “West”, “South” and “Central+East” delineated by SAMOVA analysis. **B**) **Genetic Map by Group displays relative size (** ***n*** **_individuals_) and haplotype composition of population samples within each group** (each pie slice represents one haplotype); bicolored pie charts and gray text indicate admixed populations. **C**) **SplitsTree Neighbor-Net** illustrates relative distance between haplotype groups based on uncorrected pairwise genetic distance. Each line represents the connection between two haplotypes with the thicker lines representing many connections.

**Figure 3:**
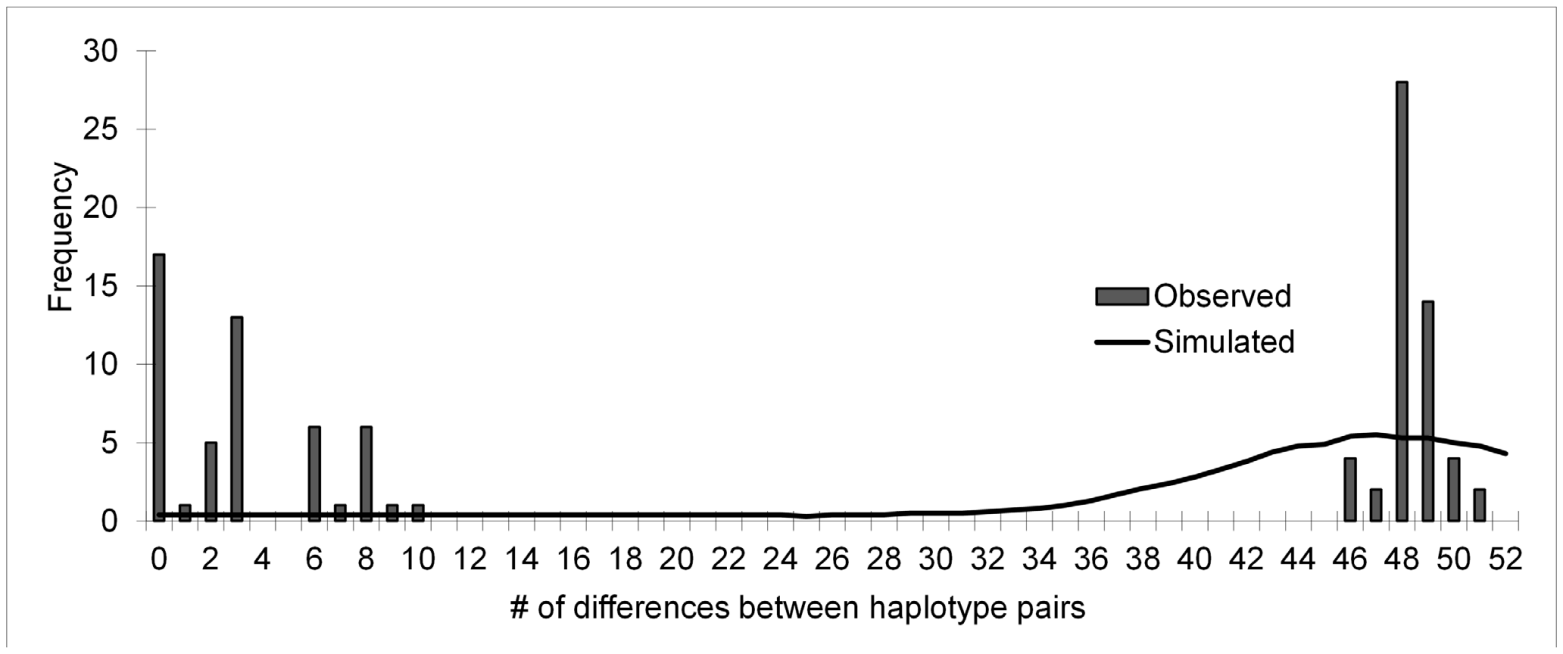
Mismatch of ND4 haplotypes within population of Java. Characteristic bimodal distribution of haplotype relationships within admixed populations.

A Bayesian phylogenetic analysis (model = K80+G) of the ND4 haplotype dataset (*n*_haplo_ = 57) revealed two deeply divergent and geographically-concordant evolutionary lineages on the island (4.3% minimum uncorrected pairwise genetic distance separating nodes A and B of Bayesian phylogram, Figure 2a). SAMOVA returned five distinct genetic groups, reflecting four geographic lineages – “North” comprising clade A, and “West”, “South” and “Central+East” representing substructure within clade B – which were clearly distinguished in the Bayesian phylogram as well as in haplotype groupings generated in SplitsTree by Neighbor-Network analyses (Figure 2c). This haplotype network, depicting the relative strength of support for pairwise relationships across all ND4 sequences produced the same four geographically-concordant groupings referenced above, and showed that the deepest divergence lay between the North and all other groups, with North most closely related to the Western group (minimum pairwise genetic distance N vs. W: 4.3%), followed by the two Western off-shoots: the Southern group (N vs. S: 5.3%) and the Central+Eastern group (N vs. C+E: 5.2%) (Figure 2c). The Western group, Central+Eastern group and Southern group were much more closely related to each other than they were to the North (≤2.0% minimum pairwise genetic distance between all two-way comparisons of these groups). In addition, SAMOVA recognized a fifth group composed of four populations of haplotypes that derived from both the Northern and the Central+Eastern lineages: Macambrara, Java, Abade+West Abade, and Santa Luzia (*e.g*., “Admixed,” Figure 2a,b). The admixed nature of these four populations was evidenced by bimodal distributions of mismatch analyses (Figure 3), reflecting the paucity of intermediate haplotypes in these “boundary” populations.

**Figure 4:**
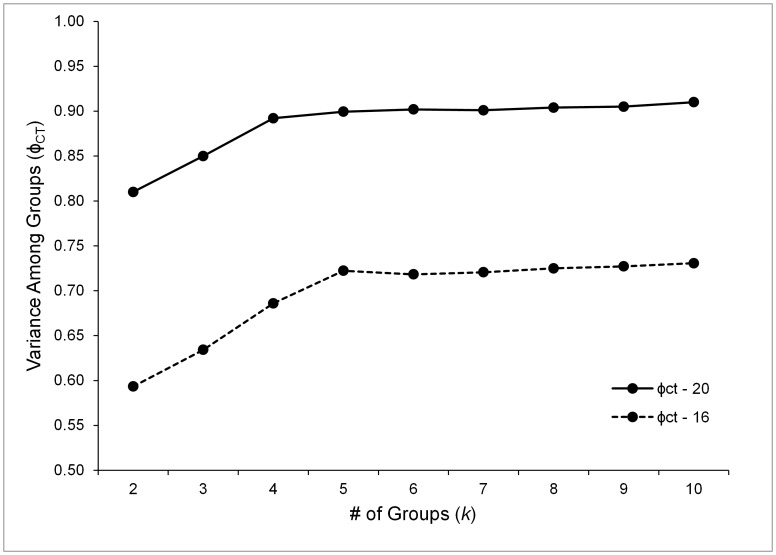
SAMOVA results illustrating variance in ND4 haplotype diversity attributable to *S. thomense* group structure (*φ*_CT_). *φ*_CT_ – 16 illustrates results for initial SAMOVA dataset, in which admixed populations are not split according to haplotype lineage: *k* = 5 groups explain almost all variation (North, West, Central+East, South, and Admixed). *φ*_CT_ – 20 illustrates results for second SAMOVA dataset, in which admixed populations are split according to haplotype lineage: *k* = 4 groups explain almost all variation (North, West, Central+East, and South).

In order to provide a more historical interpretation of matrilineal populations, a second SAMOVA was run, for which the four admixed populations were split into their component Northern and Central+Eastern subsets. As expected, this analysis identified the Northern, Western, Central+Eastern, and Southern groups, generating a *φ*_CT_ of 0.892 and explaining nearly 90% of the genetic variation in the island sample. Despite the fact that 79% of haplotypes in our dataset occurred as private alleles – reflected in a very high *φ*_ST_ (0.937) – within population variance explained only 6.34% of the total island variance. Likewise, variance among populations within groups – described by *φ*_SC_, which was lower (0.412) – explained only 4.45% of total island variance (all *p*-values<0.00001). We found *φ*_CT_ for this SAMOVA continued to rise marginally (<0.01) when the number of possible groups was increased, but adding groups did not significantly change *φ*_CT_ (Figure 4). As illustrated by the *φ*-statistics, genetic variation appeared to be highly substructured on the island. A Mantel test comparing pairwise *F*_st_ and Euclidean distance measures across populations within groups found significant evidence of isolation by distance in the Northern group (correlation coefficient = 0.36, *p* = 0.01), but not for the Central+Eastern group.

Within groups, genetic diversity – as measured by haplotype diversity (*h*) and nucleotide diversity (*π*) – was highest in the Western and Northern lineages, slightly less variable in the Central+Eastern lineage, and least variable in the Southern lineage (Table 1). The relatively high diversity in the Western lineage was notable, given its low sample size and representation by only two collection locales. The Southern lineage, in contrast, composed of similar sample size but only one locale, had considerably less diversity; each of these groups made up <10% of the total dataset. High observed diversity paired with its relative placement on the Bayesian phylogram indicated that the Western lineage was the first to diverge of the three identified on the southern half of the island (Table 1 and Figure 2a).

**Table 1:** Haplotype (*h*) and nucleotide diversity (*π*) ± standard error for four genetic groups of *Schistometopum thomense* identified from 20 sampling sites on the island of São Tomé.

	North	Central+East	West	South
***n***	80	31	13	14
**ND4 haplotypes**	31	13	9	4
**Haplotype Diversity (** ***h*** **)**	0.93±0.02	0.84±0.05	0.95±0.04	0.58±0.14
**Nucleotide Diversity (** ***π*** **)**	0.0060±0.0033	0.0034±0.0020	0.0042±0.0026	0.0013±0.0010

Nucleotide diversity estimates represent the average number of nucleotide differences between two sequences chosen at random [70]. Haplotype diversity estimates represent the likelihood of randomly selecting two different haplotypes from a sample [69].

IMa2 analysis of a composite ND4 and 16S dataset (*n*_individuals_ = 26, mutation rate16S: 2.161029, mutation rate ND4: 5.761029) allowed greater insight into lineage ages. Two distinct divergence periods were identified: one between 200–250 Kya and the other between 20–30 Kya. The first divergence between Northern and other lineages produced the highest point for divergence (HPD) at 253 Kya (95% HPD = 136–435 Kya), and the lineage on the west coast appears to have split very close to this time (average HPD = 215 Kya: 95% HPD = 101–354 Kya). Most recently, the lineage in the extreme south diverged from the lineage in the central and eastern areas around 27 Kya (95% HPD = 2–242 Kya). Although 95% HPDs were overlapping for all of these events, the highest point for the HPD for each of the divergence events was used as an indication of the most likely divergence scenario.

Evidence for recent population expansion in the Northern lineage was obtained in results of mismatch analysis, Fu's *F*_s_ and Tajima's *D* (mismatch Sum of Squared Difference = 0.0078, *p*-value>0.5 and Harpending's Raggedness = 0.021, *p*-value>0.3; Fu's *F_S_* = −13.08, *p* = 0.0012; Tajima's D = −1.80, *p*-value = 0.0121), allowing an additional calculation of time since the inferred demographic expansion in the North of São Tomé: ca. 167.5 Kya (based on *τ* = 2.566, 95% CI: 0.182–15.035, which estimated *t* = 83,747 generations and used a generation time of 2 years). The Central+Eastern and Western groups also showed conformity to a pattern of recent population expansion based on mismatch distribution tests, but for neither of these groups were Tajima's *D* or Fu's *F*_s_ significantly negative. The Bayesian phylogram shows the same relative ‘ages’ of the lineages and the haplotypes within: apparent ancestral haplotypes were collected from the west of the island (Haplotype 25 from Rio Maria Luisa [Northern lineage], and Haplotypes 54 and 15 from Lemba and Binda, respectively [Western lineage], Figure 2a,b).

### Color

**Figure 5:**
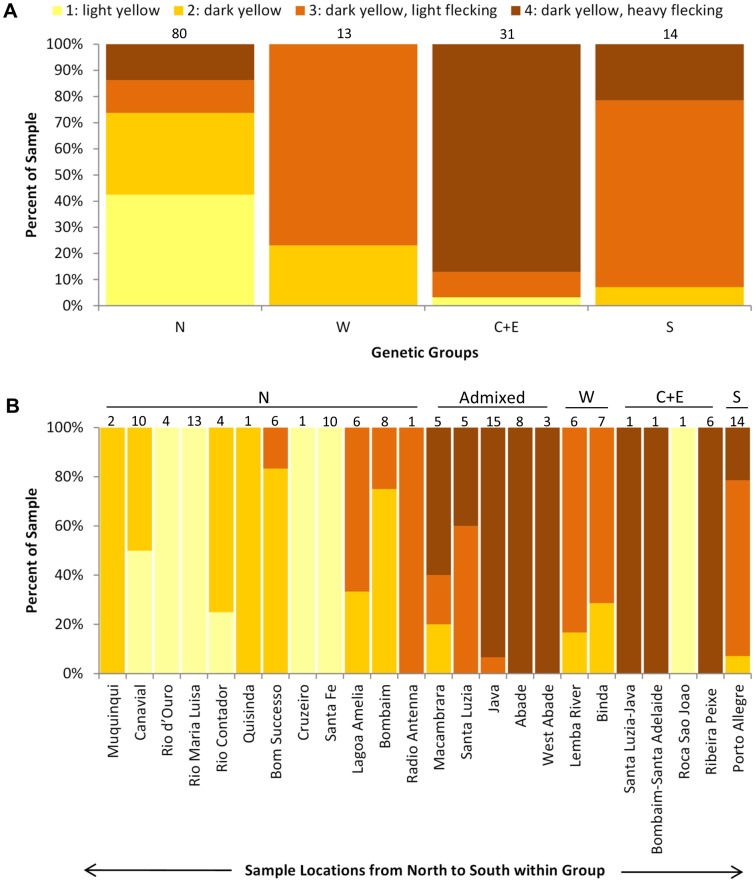
Color categorization of specimens based on Nussbaum and Pfrender's [36] ventral color descriptions. **A) By haplotype group, and B) By collection locality.** Numbers above bars are sample sizes (*n*_total_ = 138). N = North, C+E = Central+East, W = West, S = South, and Admixed = admixed populations containing haplotypes in both the Northern and Central+Eastern genetic groups.

**Figure 6:**
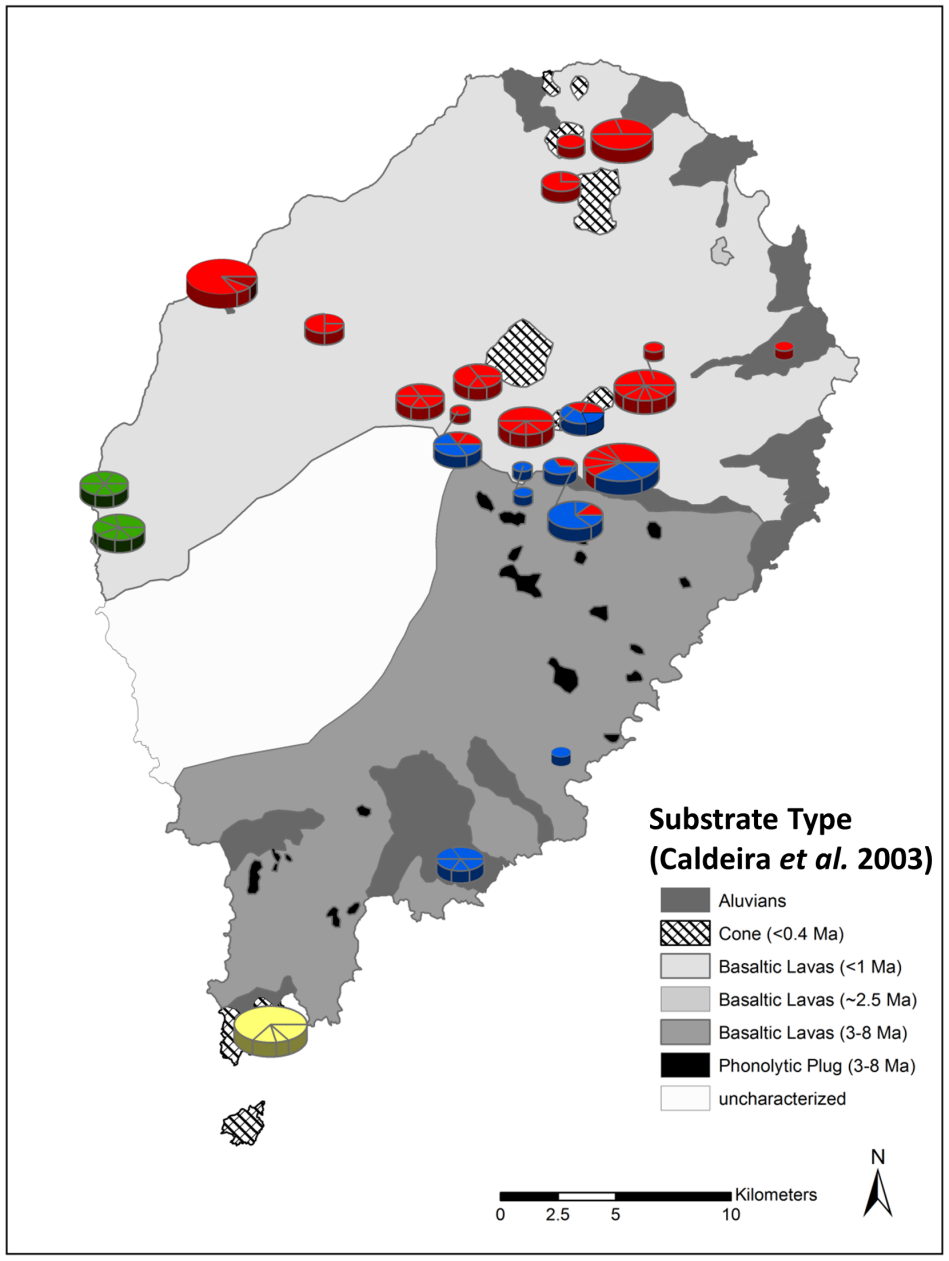
Distribution of haplotype groups in relation to island volcanism. Geologic data borrowed from Figure 1 of Caldeira et al. [21].

Dorsal coloration was mixed within and across all genetic groups, but, on average, animals from the Northern collection localities were lightest and animals from the Central+Eastern collection localities were darkest (Figure 5a, for categories 1 through 4, *n*_1_ = 35, *n*_2_ = 29, *n*_3_ = 33, *n*_4_ = 41). Our AIC_c_ ordering of multinomial logistic regressions selected the global model – retaining the explanatory variables of genetic group, geology, latitude and altitude – as the best model (AIC_c_ = 195.85, k = 27), ranking more than 21 AIC_c_ units above the next best model and accounting for virtually all the weight among our candidate model set. Despite retaining all explanatory variables, the best model only produced significant Type III effects for genetic group (*F*_9,111_ = 2.3, *p* = 0.02) and latitude (*F*_3,111_ = 3.1, *p* = 0.03). Examination of 95% confidence limits surrounding *β* estimates indicated that most support for these covariates came from the comparison between dark yellow, unflecked morphs (category 2) to dark yellow, flecked morphs (category 4; Table 2); these unflecked morphs were less likely to have haplotypes in the Central+Eastern group than in the Western group and more likely to have been collected at higher latitudes. They also were less likely to have been collected on basaltic flows <1 Mya than on younger pyroclastic lava cones, and, in general, were less likely to occur in the dataset than the darker, flecked individuals (intercept term “2/4”; Table 2). Latitude similarly affected the relative occurrence of light yellow, unflecked morphs (category 1) to that of the dark yellow, flecked morphs; category 1 morphs were more likely to be found at higher latitudes (Table 2). Overall, given the size of our dataset (*n* = 138) and number of individuals in each response category (*n*_color_ = ∼35) relative to total model parameters (k = 27), our ability to detect significant effects may have been restricted. For the geology covariate, sample size may have limited the power to detect an effect in some levels, e.g., only one specimen resided on older basaltic lava flows 3–8 Mya (Figure 6). Further observations from raw data showed that within populations of *n*≥2, substantial variation existed; more than 25% of the populations contained both clear and flecked individuals (Figure 5b). Of the seven collection locales that contained clear and flecked individuals, only one was genetically admixed (Macambrara); the remainder were uniquely Northern, Western, or Southern. We expect that investigation of nDNA patterns in these and the other admixed populations will clarify questions that remain about the interplay of color morphology and genotype for these caecilians.

**Table 2:** Regression coefficients (odds ratios) and 95% confidence limits from top AIC*_c_*-ranked model returned by multinomial logistic regression of dorsal color on genetic group, latitude, altitude and geology.

	model coefficients (odds ratios)	*β* estimate	95% CL
**intercepts**	1/4	−31.89	−68.79, 5.01
	2/4	−32.95*	−64.97, −0.93
	3/4	−17.16	−49.73, 15.42
	alluvial/cone (1/4)	9.89	−51.82, 71.59
	alluvial/cone (2/4)	12.49	−48.57, 73.64
	alluvial/cone (3/4)	11.99	−48.53, 72.50
**geology**	basalt 3–8 Mya/cone (1/4)	21.87	−34.46, 78.31
	basalt 3–8 Mya/cone (2/4)	27.19	−87.37, 141.75
	basalt 3–8 Mya/cone (3/4)	15.42	−78.32, 109.16
	basalt <1 Mya/cone (1/4)	2.61	−4.90, 10.11
	basalt <1 Mya/cone (2/4)	−3.84*	−7.45, −0.23
	basalt <1 Mya/cone (3/4)	−3.50	−7.07, 0.07
**latitude**	1/4	126.07*	11.21, 240.94
	2/4	175.41*	60.69, 290.14
	3/4	113.22	−2.11, 228.55
**altitude**	1/4	−0.03	−0.05, 0.00
	2/4	0.00	−0.00, 0.01
	3/4	0.00	−0.00, 0.01
	C+E/W (1/4)	1.94	−25.32, 29.21
	C+E/W (2/4)	−20.89*	−40.73, −1.05
	C+E/W (3/4)	−15.52	−32.52, 1.48
**genetic group**	N/W (1/4)	6.21	−19.69, 32.10
	N/W (2/4)	−14.24	−31.32, 2.85
	N/W (3/4)	−13.37	−30.18, 3.45
	S/W (1/4)	22.77	−14.53, 60.07
	S/W (2/4)	25.10	−3.42, 53.62
	S/W (3/4)	13.97	−15.03, 42.96

Odds ratios are interpreted as the likelihood of being in a particular category in reference to a baseline (e.g., for the model coefficient “geology: alluvial/cone (1/4),” the *β* estimate represents the probability of being in color category 1 versus 4 given that animal was collected on alluvial soil versus a lava cone). Significant *β* estimates are indicated by an asterisk.

## Discussion

Our results suggest that both genetic clades and clinal variation contribute to the strikingly high differences in coloration that led Taylor [34] to describe *Schistometopum “ephele”* as a distinct taxon from *Schistometopum thomense* on the island of São Tomé. Nussbaum and Pfrender [36] showed the importance of increased sampling in their work aimed at unraveling historic discrepancies in *Schistometopum* taxonomy. In contrast to their results, which identified a morphological cline only, our wider sampling (especially in the North), coupled with genetic data, show that *S. thomense*, as currently recognized, is highly structured for mtDNA and that this structure is concordant with northern, western, eastern+central and southern geographic groupings on the island. Although, a north-south cline is not rejected by our morphological analyses (i.e., inclusion of the latitude covariate in our best multinomial logistic regression), our mitochondrial genetic results show that this species exhibits a narrow zone of admixture between the two most divergent clades (∼4% minimum uncorrected pairwise distance between ND4 lineages) and ages this split at approximately 0.25 Mya. We require nDNA sequencing to ascertain whether the admixed area contains hybrids between the two mtDNA lineages. However, the mixed nature of color forms in these areas suggests that this is the case. Admixed zones have been found for other amphibians which have arrived on islands [87], and for natural populations in a ring species [88], and this challenges the traditional view of genetic equilibrium in population genetics. In our example from the island of São Tomé, we suggest that environmental disturbance (volcanism) is capable of producing these highly structured groupings.

Whether *S. thomense* arrived on São Tomé by a single or multiple colonization events remains inconclusive, given the relatively recent estimates of population divergence (relative to island age) and of volcanic flows. Multiple independent colonization events have been suggested for geckos [22]–[23], skinks [24] and various snake species [25]. Despite an approximate (sub-aerial) age of 13 Mya for the island [18], two of these studies have produced remarkably recent dates for colonizations and radiations on the island: ca 800 Kya and ca 200 Kya for skinks [24], and ca 800 Kya and ca 600 Kya for passerine birds (*Zosterops* spp. and *Speirops* spp., respectively) [89]. As pointed out by Jesus et al. [24], volcanic activity may be responsible for these recent dates, either having wiped out evidence of earlier colonizations or having provided impetus for diversification.

The most recent eruptions have been placed at 36 Kya, in geologic samples collected from the islet of Rolas (immediately South of our most southerly collection locale, Porto Allegre on São Tomé) [19]. This date is close to our most recent estimated date for population divergence which also corresponds to the split between the southern population, and the eastern population (of which the next nearest sampling locale was Ribeira Peixe, approximately 11 km northeast). We infer this corroboration in dates defining population groups as an indication that volcanism on São Tomé is the likely cause of other genetic groups that we define. The northern population group corresponds well with the <1Ma northern lava flow (Figure 5). The geological map shows that the North of the island contains numerous small pockets along the coast which could have acted as refugia. This scenario also corresponds with the estimated date of expansion of this northern population. Interestingly, the admixed populations occur almost exactly on the borders of this area suggesting recolonization from southern populations was limited although the reason for this is unclear. Although we cannot rule out that genetic groups may represent separate colonization events, especially in light of an apparently ideal delivery system [27], we speculate that volcanic activity is capable of producing these genetic patterns. Alternatively, our results could be explained by a combination of volcanic activity (Central+East, West and South) and multiple colonization events (North and all others: Central+East, West and South); i.e., both taxonomic and clinal variation. Sampling of the elusive taxon from the Democratic Republic of the Congo may prove critical in eliminating this hypothesis.

Amphibians have been classically described as highly philopatric [90], with strong population structuring over relatively small spatial scales [91]–[92]. Caecilians, in particular, have been considered poor dispersers because of their fossorial nature and sensitivity to the moisture content of surrounding environments [93]–[94]. However, the only published study of intraspecific caecilian genetics (*Ichthyophis* sp. from the Western Ghats of India) suggests that caecilians are not necessarily philopatric [95], although a more recent study from Southeast Asia suggests much higher structuring over a far smaller area [96]. The strong population genetic structure shown in our study, together with the large number of private alleles, is therefore more in line with this and the traditional view of amphibians, and is somewhat extreme given the small size of the island and almost ubiquitous occupancy of this species on São Tomé (RCD, GJM & RES *pers. obs.*). An explanation for these two very different results for caecilian taxa may stem from the Indian study being based on a very small sample (*n* = 18) using relatively undifferentiated mtDNA markers (12S and 16S) in a phylogeographic context [95]. Our study is therefore, the first population genetic study of any caecilian and we suggest that our results showing strong genetic structuring over very small spatial scales may be represented more widely by many terrestrial gymnophiones. Our results have important implications for conservation of caecilians, especially where populations have become fragmented.

We emphasize provisional acceptance of our results given potential bias posed by use of a two-gene mtDNA dataset. Because (1) natural selection influences mutations in functional genes, (2) the coalescence of gene lineages to cladogenic events may be slow to reflect organismal phylogeny or gene lineages may diverge within a taxonomic line and insinuate a taxonomic division when one does not exist, and (3) – for most taxa – mitochondrial phylogenies track only the evolutionary history of matriarchal lines, we highlight the fact that cladogenic results reported here require substantiation with nDNA. However, we note that single-gene mtDNA histories have proven informative in many studies [97]–[105] and that our dataset appears to be an appropriate information source for *S. thomense* on São Tomé given the neutral behavior of the mutations observed, the geographic congruence of haplotype pattern, and the generalized concordance with morphological data.

## Conclusions

*Schistometopum thomense* on São Tomé has a strongly differentiated population structure shown by mtDNA, something that was hitherto unknown for any species in this amphibian Order. Our study confirms previous hypotheses concerning clinal latitudinal variation [36] and philopatry [38], but was inconclusive about the importance of color for crypsis [84] using our geological proxy for soil type and vegetation type. Taxonomically, our results were inconclusive because we could not investigate a clear zone of admixture present on the island between two deeply divergent clades (which would correspond to *S. thomense* and *S. ephele*), although applying genetic barcoding standards to 16S [82], [106], our 4.3% divergence would not be considered deep enough to warrant taxonomic differentiation. Further investigation into the admixed zone is required with nDNA to provide more conclusive genetic results.

## Supporting Information

Abstract S1 Resumo. Abstract in Portuguese.
(DOCX)

Table S1 Specimen Data.
(DOCX)
