# Correction: Population Genetics of the São Tomé Caecilian (Gymnophiona: Dermophiidae: *Schistometopum thomense*) Reveals Strong Geographic Structuring

**DOI:** 10.1371/journal.pone.0116005

**Published:** 2014-12-15

**Authors:** The PLOS ONE Staff

The parameter name in the Materials and Methods section and Results section are incorrectly formatted. In all cases, the correct parameter name is Fu’s *F_s_*.

The genetic variability measure in the Results section is incorrectly formatted. The correct genetic variability measure is *F*_ST._

The Figure 1 legend is incorrect. The word “Red” should be “Black” in the last sentence. The authors have provided a corrected version here.

**Figure 1:**
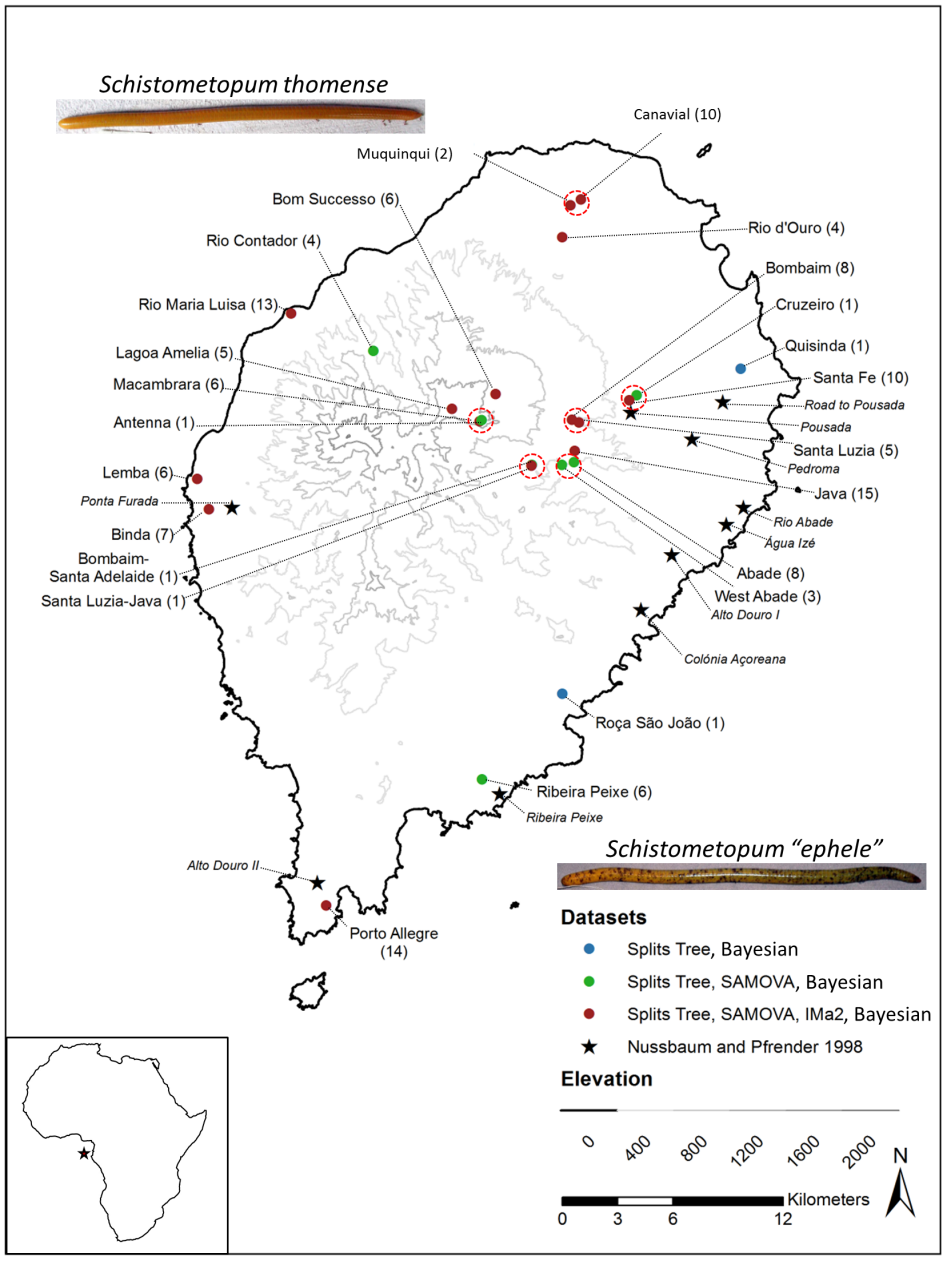
*Schistometopum thomense* collection localities, São Tomé, Republic of São Tomé and Príncipe. Numbers in parentheses indicate number of specimens available for genetic analyses. Legend indicates specimens used in Splits Tree, Bayesian, SAMOVA, and/or IMa2 analyses, or discussed by Nussbaum and Pfrender [36] in morphological comparisons. Dashed, red ovals indicate populations lumped for SAMOVA analyses. Photographs show examples of clear and flecked morphs as *Schistometopum thomense* and Schistometopum “ephele”, respectively. Red star in lower left panel indicates relative position of São Tomé to continental Africa.
