# Supplementary material for: Population Genetics of the São Tomé Caecilian (Gymnophiona: Dermophiidae: Schistometopum thomense) Reveals Strong Geographic Structuring
Source: PLoS One. 2014 Aug 29;9(8):e104628. doi: 10.1371/journal.pone.0104628 (PMC4149351; doi:10.1371/journal.pone.0104628)
Supplement: Abstract S1 — Resumo. Abstract in Portuguese. (DOCX) [file pone.0104628.s001.docx]

**Resumo** *(Abstract in Portuguese)*
Ilhas oferecem grandes oportunidades para explorar mecanismos ecológicos e evolutivos devido ao seu isolamento. A ilha oceânica de São Tomé, no Golfo Africano Ocidental da Guiné, tornou-se de especial interesse devido à sua alta diversidade de anfíbios, incluindo o endêmico anfíbio caecilian, *Schistometopum thomense*. Impressionante variação na pigmentação amarela, morfologia e tamanho além dos aproximadamente 45 km de ilha é extremo e tem levado a uma série de hipóteses taxonômicas, ecológicas e evolutivas. Realizamos um estudo de genética de populações desta espécie utilizando sequências parciais de dois genes mitocondriais (ND4 e 16S) juntamente com dados morfológicos usando hipóteses concorrentes no taxonômico ou variação clinal para explicar as diferenças na espécie. Usando a análise filogenética Bayesiana, análise espacial de Variância Molecular (SAMOVA) e encontramos evidências de quatro clados geográficos cujo alcance e tempo para o ancestral comum mais recente (aproximadamente 253 Kya - 27 Kya, dependendo da divisão: do isolamento com análises migratória) são consistentes com propagação e idade dos últimos fluxos vulcânicos da ilha. Estes grupos genéticos explicam mais de 90% de variação na ND4 (*ɸ_CT_* = 0,892), e divergem no mínimo de 4,3% em divergência genética entre o nó dos mais profundo clados. Além disso, usando distribuições de incompatibilidade e testes de Mantel, identificamos uma zona de mistura população que disseca a ilha, bem como evidências de expansão populacional recente (de Fu *F_s_* = -13,08 e de Tajima *D* = -1,80) e dispersão limitada (coeficiente de correlação de Mantel = 0,36, *p* = 0,01) no Norte para este taxon. Emparelhado com regressão multinomial de dados cromáticas, no entanto, nossas análises genéticas sugerem que os grupos genéticos e um gradiente latitudinal juntos descrevem variações na cor de *Schistometopum* na ilha, e que o vulcanismo e limitada capacidade de dispersão são as causas prováveis ​da estrutura genética observada. Este é o primeiro estudo genético de população de caecilian e demonstra que estes animais têm profundas divisões genéticas em áreas muito pequenas de acordo com especulações anteriores de baixas capacidades de dispersão.
