# Supplementary material for: Population Genetics of the São Tomé Caecilian (Gymnophiona: Dermophiidae: Schistometopum thomense) Reveals Strong Geographic Structuring
Source: PLoS One. 2014 Aug 29;9(8):e104628. doi: 10.1371/journal.pone.0104628 (PMC4149351; doi:10.1371/journal.pone.0104628)
Supplement: Table S1 — Specimen Data. (DOCX) [file pone.0104628.s002.docx]

**Table S1. Specimen Data.**

| **Collection** | **Specimen No.** | **Tissue No.** | **Locality** | **Latitude** | **Longitude** | **Scientific Name** | **IMA Bayesian Prior** |  |  |  |  |  |  |
| --- | --- | --- | --- | --- | --- | --- | --- | --- | --- | --- | --- | --- | --- |
| JM | 00045 | JM00046 | Canavial | 0.384028 | 6.653972 | *Schistometopum thomense* |  |  |  |  |  |  |  |
| JM | 00047 | JM00048 | Canavial | 0.384028 | 6.653972 | *Schistometopum thomense* | *X* |  |  |  |  |  |  |
| JM | 00049 | JM00050 | Canavial | 0.384028 | 6.653972 | *Schistometopum thomense* |  |  |  |  |  |  |  |
| JM | 00051 | JM00052 | Canavial | 0.384028 | 6.653972 | *Schistometopum thomense* |  |  |  |  |  |  |  |
| JM | 00053 | JM00054 | Canavial | 0.384028 | 6.653972 | *Schistometopum thomense* |  |  |  |  |  |  |  |
| BMNH | 2000.336 | JM00091 | Porto Allegre | 0.035722 | 6.531389 | *Schistometopum thomense* |  |  |  |  |  |  |  |
| JM | 00077 | JM00092 | Porto Allegre | 0.035722 | 6.531389 | *Schistometopum thomense* | *X* |  |  |  |  |  |  |
| BMNH | 2000.334 | JM00093 | Porto Allegre | 0.035722 | 6.531389 | *Schistometopum thomense* |  |  |  |  |  |  |  |
| BMNH | 2000.333 | JM00094 | Porto Allegre | 0.035722 | 6.531389 | *Schistometopum thomense* |  |  |  |  |  |  |  |
| BMNH | 2000.332 | JM00095 | Porto Allegre | 0.035722 | 6.531389 | *Schistometopum thomense* |  |  |  |  |  |  |  |
| BMNH | 2000.329 | JM00096 | Porto Allegre | 0.035722 | 6.531389 | *Schistometopum thomense* |  |  |  |  |  |  |  |
| BMNH | 2000.330 | JM00097 | Porto Allegre | 0.035722 | 6.531389 | *Schistometopum thomense* |  |  |  |  |  |  |  |
| BMNH | 2000.335 | JM00098 | Porto Allegre | 0.035722 | 6.531389 | *Schistometopum thomense* |  |  |  |  |  |  |  |
| BMNH | 2000.338 | JM00099 | Porto Allegre | 0.035722 | 6.531389 | *Schistometopum thomense* |  |  |  |  |  |  |  |
| BMNH | 2000.340 | JM00100 | Porto Allegre | 0.035722 | 6.531389 | *Schistometopum thomense* |  |  |  |  |  |  |  |
| JM | 00395 | JM00396 | Ribiera Peixe | 0.100444 | 6.605583 | *Schistometopum thomense* |  |  |  |  |  |  |  |
| JM | 00397 | JM00398 | Ribiera Peixe | 0.100444 | 6.605583 | *Schistometopum thomense* |  |  |  |  |  |  |  |
| JM | 00601 | JM00602 | Ribiera Peixe | 0.100444 | 6.605583 | *Schistometopum thomense* |  |  |  |  |  |  |  |
| JM | 00603 | JM00604 | Ribiera Peixe | 0.100444 | 6.605583 | *Schistometopum thomense* |  |  |  |  |  |  |  |
| JM | 00605 | JM00606 | Ribiera Peixe | 0.100444 | 6.605583 | *Schistometopum thomense* |  |  |  |  |  |  |  |
| JM | 00607 | JM00608 | Ribiera Peixe | 0.100444 | 6.605583 | *Schistometopum thomense* |  |  |  |  |  |  |  |
| JM | 00664 | JM00668 | Macambrara | 0.275472 | 6.604500 | *Schistometopum thomense* |  |  |  |  |  |  |  |
| JM | 00665 | JM00669 | Macambrara | 0.275472 | 6.604500 | *Schistometopum thomense* | *X* |  |  |  |  |  |  |
| JM | 00666 | JM00670 | Macambrara | 0.275472 | 6.604500 | *Schistometopum thomense* |  |  |  |  |  |  |  |
| JM | 00671 | JM00674 | Macambrara | 0.275472 | 6.604500 | *Schistometopum thomense* |  |  |  |  |  |  |  |
| JM | 00672 | JM00675 | Macambrara | 0.275472 | 6.604500 | *Schistometopum thomense* | *X* |  |  |  |  |  |  |
| JM | 00673 | JM00676 | Macambrara | 0.275472 | 6.604500 | *Schistometopum thomense* |  |  |  |  |  |  |  |
| JM | 00677 | JM00678 | Radio Antenna | 0.276062 | 6.605522 | *Schistometopum thomense* | *X* |  |  |  |  |  |  |
| JM | 00701 | JM00704 | Binda | 0.232417 | 6.472194 | *Schistometopum thomense* |  |  |  |  |  |  |  |
| JM | 00702 | JM00705 | Binda | 0.232417 | 6.472194 | *Schistometopum thomense* | *X* |  |  |  |  |  |  |
| JM | 00703 | JM00706 | Binda | 0.232417 | 6.472194 | *Schistometopum thomense* |  |  |  |  |  |  |  |
| JM | 00698 | JM00707 | Binda | 0.232417 | 6.472194 | *Schistometopum thomense* |  |  |  |  |  |  |  |
| JM | 00699 | JM00708 | Binda | 0.232417 | 6.472194 | *Schistometopum thomense* | *X* |  |  |  |  |  |  |
| JM | 00700 | JM00709 | Binda | 0.232417 | 6.472194 | *Schistometopum thomense* |  |  |  |  |  |  |  |
| JM | 00710 | JM00713 | Binda | 0.232417 | 6.472194 | *Schistometopum thomense* | *X* |  |  |  |  |  |  |
| JM | 00749 | JM00752 | Lagoa Amelia | 0.281583 | 6.590889 | *Schistometopum thomense* |  |  |  |  |  |  |  |
| JM | 00750 | JM00753 | Lagoa Amelia | 0.281583 | 6.590889 | *Schistometopum thomense* | *X* |  |  |  |  |  |  |
| JM | 00757 | JM00758 | Lagoa Amelia | 0.281583 | 6.590889 | *Schistometopum thomense* |  |  |  |  |  |  |  |
| JM | 00759 | JM00760 | Lagoa Amelia | 0.281583 | 6.590889 | *Schistometopum thomense* |  |  |  |  |  |  |  |
| JM | 00761 | JM00762 | Lagoa Amelia | 0.281583 | 6.590889 | *Schistometopum thomense* |  |  |  |  |  |  |  |
| BMNH | 2000.32 | JM00042 | Canavial | 0.384028 | 6.653972 | *Schistometopum thomense* |  |  |  |  |  |  |  |
| BMNH | 2000.321 | JM00044 | Canavial | 0.384028 | 6.653972 | *Schistometopum thomense* | *X* |  |  |  |  |  |  |
| BMNH | 2000.323 | JM00060 | Canavial | 0.384028 | 6.653972 | *Schistometopum thomense* |  |  |  |  |  |  |  |
| CAS | 218731 | NA | Java | 0.261083 | 6.650889 | *Schistometopum thomense* | *X* |  |  |  |  |  |  |
| CAS | 218732 | NA | Java | 0.261083 | 6.650889 | *Schistometopum thomense* |  |  |  |  |  |  |  |
| CAS | 218733 | NA | Java | 0.261083 | 6.650889 | *Schistometopum thomense* |  |  |  |  |  |  |  |
| CAS | 218734 | NA | Java | 0.261083 | 6.650889 | *Schistometopum thomense* |  |  |  |  |  |  |  |
| CAS | 218735 | NA | Java | 0.261083 | 6.650889 | *Schistometopum thomense* |  |  |  |  |  |  |  |
| CAS | 218736 | NA | Java | 0.261083 | 6.650889 | *Schistometopum thomense* |  |  |  |  |  |  |  |
| CAS | 218737 | NA | Java | 0.261083 | 6.650889 | *Schistometopum thomense* |  |  |  |  |  |  |  |
| CAS | 218738 | NA | Java | 0.261083 | 6.650889 | *Schistometopum thomense* |  |  |  |  |  |  |  |
| CAS | 218739 | NA | Java | 0.261083 | 6.650889 | *Schistometopum thomense* | *X* |  |  |  |  |  |  |
| CAS | 218740 | NA | Java | 0.261083 | 6.650889 | *Schistometopum thomense* | *X* |  |  |  |  |  |  |
| CAS | 218741 | NA | Java | 0.261083 | 6.650889 | *Schistometopum thomense* | *X* |  |  |  |  |  |  |
| CAS | 218742 | NA | Java | 0.261083 | 6.650889 | *Schistometopum thomense* |  |  |  |  |  |  |  |
| CAS | 218743 | NA | Java | 0.261083 | 6.650889 | *Schistometopum thomense* |  |  |  |  |  |  |  |
| CAS | 218744 | NA | Java | 0.261083 | 6.650889 | *Schistometopum thomense* |  |  |  |  |  |  |  |
| CAS | 218745 | NA | Java | 0.261083 | 6.650889 | *Schistometopum thomense* |  |  |  |  |  |  |  |
| CAS | 218773 | NA | between Santa Luzia and Java at bridge over Agua Panada | 0.253890 | 6.629810 | *Schistometopum thomense* | *X* |  |  |  |  |  |  |
| CAS | 218774 | NA | Santa Luzia | 0.274972 | 6.653028 | *Schistometopum thomense* |  |  |  |  |  |  |  |
| CAS | 218775 | NA | Santa Luzia | 0.274972 | 6.653028 | *Schistometopum thomense* |  |  |  |  |  |  |  |
| CAS | 218776 | NA | Santa Luzia | 0.274972 | 6.653028 | *Schistometopum thomense* | *X* |  |  |  |  |  |  |
| CAS | 218777 | NA | Santa Luzia | 0.274972 | 6.653028 | *Schistometopum thomense* |  |  |  |  |  |  |  |
| CAS | 218778 | NA | Santa Luzia | 0.274972 | 6.653028 | *Schistometopum thomense* | *X* |  |  |  |  |  |  |
| CAS | 218794 | NA | Rio Maria Luisa | 0.32825 | 6.512333 | *Schistometopum thomense* | *X* |  |  |  |  |  |  |
| CAS | 218795 | NA | Rio Maria Luisa | 0.32825 | 6.512333 | *Schistometopum thomense* |  |  |  |  |  |  |  |
| CAS | 218796 | NA | Rio Maria Luisa | 0.32825 | 6.512333 | *Schistometopum thomense* |  |  |  |  |  |  |  |
| CAS | 218797 | NA | Rio Maria Luisa | 0.32825 | 6.512333 | *Schistometopum thomense* | *X* |  |  |  |  |  |  |
| CAS | 218798 | NA | Rio Maria Luisa | 0.32825 | 6.512333 | *Schistometopum thomense* |  |  |  |  |  |  |  |
| CAS | 218799 | NA | Rio Maria Luisa | 0.32825 | 6.512333 | *Schistometopum thomense* |  |  |  |  |  |  |  |
| CAS | 218800 | NA | Rio Maria Luisa | 0.32825 | 6.512333 | *Schistometopum thomense* |  |  |  |  |  |  |  |
| CAS | 218801 | NA | Rio Maria Luisa | 0.32825 | 6.512333 | *Schistometopum thomense* |  |  |  |  |  |  |  |
| CAS | 218802 | NA | Rio Maria Luisa | 0.32825 | 6.512333 | *Schistometopum thomense* |  |  |  |  |  |  |  |
| CAS | 218803 | NA | Rio Maria Luisa | 0.32825 | 6.512333 | *Schistometopum thomense* |  |  |  |  |  |  |  |
| CAS | 218903 | NA | between Bombaim and Santa Adelaide at Rio Abade bridge | 0.254167 | 6.630028 | *Schistometopum thomense* |  |  |  |  |  |  |  |
| CAS | 218914 | NA | Rio Contador | 0.309917 | 6.552444 | *Schistometopum thomense* |  |  |  |  |  |  |  |
| CAS | 218915 | NA | Rio Contador | 0.309917 | 6.552444 | *Schistometopum thomense* |  |  |  |  |  |  |  |
| CAS | 218960 | NA | Muquinqui | 0.381194 | 6.648778 | *Schistometopum thomense* | *X* |  |  |  |  |  |  |
| CAS | 218961 | NA | Muquinqui | 0.381194 | 6.648778 | *Schistometopum thomense* |  |  |  |  |  |  |  |
| CAS | 219031 | NA | Rio d’Ouro | 0.365472 | 6.644917 | *Schistometopum thomense* |  |  |  |  |  |  |  |
| CAS | 219032 | NA | Rio d’Ouro | 0.365472 | 6.644917 | *Schistometopum thomense* |  |  |  |  |  |  |  |
| CAS | 219033 | NA | Rio d’Ouro | 0.365472 | 6.644917 | *Schistometopum thomense* |  |  |  |  |  |  |  |
| CAS | 219034 | NA | Rio d’Ouro | 0.365472 | 6.644917 | *Schistometopum thomense* | *X* |  |  |  |  |  |  |
| CAS | 219074 | NA | Canavial | 0.384028 | 6.653972 | *Schistometopum thomense* |  |  |  |  |  |  |  |
| CAS | 219075 | NA | Canavial | 0.384028 | 6.653972 | *Schistometopum thomense* |  |  |  |  |  |  |  |
| CAS | 219286 | NA | Quisinda | 0.301111 | 6.732028 | *Schistometopum thomense* |  |  |  |  |  |  |  |
| CAS | 219292 | NA | Roca Sao Joao | 0.142250 | 6.644889 | *Schistometopum thomense* |  |  |  |  |  |  |  |
| CAS | 219295 | NA | Santa Fe | 0.285750 | 6.677667 | *Schistometopum thomense* | *X* |  |  |  |  |  |  |
| CAS | 219296 | NA | Santa Fe | 0.285750 | 6.677667 | *Schistometopum thomense* |  |  |  |  |  |  |  |
| CAS | 219297 | NA | Santa Fe | 0.285750 | 6.677667 | *Schistometopum thomense* |  |  |  |  |  |  |  |
| CAS | 219298 | NA | Santa Fe | 0.285750 | 6.677667 | *Schistometopum thomense* |  |  |  |  |  |  |  |
| CAS | 219299 | NA | Santa Fe | 0.285750 | 6.677667 | *Schistometopum thomense* |  |  |  |  |  |  |  |
| CAS | 219300 | NA | Santa Fe | 0.285750 | 6.677667 | *Schistometopum thomense* | *X* |  |  |  |  |  |  |
| CAS | 219301 | NA | Santa Fe | 0.285750 | 6.677667 | *Schistometopum thomense* |  |  |  |  |  |  |  |
| CAS | 219302 | NA | Santa Fe | 0.285750 | 6.677667 | *Schistometopum thomense* |  |  |  |  |  |  |  |
| CAS | 219303 | NA | Santa Fe | 0.285750 | 6.677667 | *Schistometopum thomense* |  |  |  |  |  |  |  |
| CAS | 219304 | NA | Santa Fe | 0.285750 | 6.677667 | *Schistometopum thomense* |  |  |  |  |  |  |  |
| CAS | 219312 | NA | Cruzeiro | 0.288278 | 6.681194 | *Schistometopum thomense* |  |  |  |  |  |  |  |
| CAS | 219324 | NA | Bom Successo | 0.288758 | 6.612354 | *Schistometopum thomense* |  |  |  |  |  |  |  |
| CAS | 219325 | NA | Bom Successo | 0.288758 | 6.612354 | *Schistometopum thomense* | *X* |  |  |  |  |  |  |
| CAS | 219326 | NA | Bom Successo | 0.288758 | 6.612354 | *Schistometopum thomense* |  |  |  |  |  |  |  |
| CAS | 219327 | NA | Bom Successo | 0.288758 | 6.612354 | *Schistometopum thomense* | *X* |  |  |  |  |  |  |
| CAS | 219328 | NA | Bom Successo | 0.288758 | 6.612354 | *Schistometopum thomense* |  |  |  |  |  |  |  |
| CAS | 219329 | NA | Bom Successo | 0.288758 | 6.612354 | *Schistometopum thomense* |  |  |  |  |  |  |  |
| CAS | 233451 | NA | Rio Contador at aqueduct | 0.311080 | 6.552440 | *Schistometopum thomense* | *X* |  |  |  |  |  |  |
| CAS | 233452 | NA | Rio Contador at aqueduct | 0.311080 | 6.552440 | *Schistometopum thomense* |  |  |  |  |  |  |  |
| CAS | 233454 | NA | Bombaim (9 km E) | 0.276333 | 6.649667 | *Schistometopum thomense* |  |  |  |  |  |  |  |
| CAS | 233456 | NA | Bombaim (9 km E) | 0.276333 | 6.649667 | *Schistometopum thomense* |  |  |  |  |  |  |  |
| CAS | 233457 | NA | Bombaim (9 km E) | 0.276333 | 6.649667 | *Schistometopum thomense* | *X* |  |  |  |  |  |  |
| CAS | 233458 | NA | Bombaim (9 km E) | 0.276333 | 6.649667 | *Schistometopum thomense* |  |  |  |  |  |  |  |
| CAS | 233459 | NA | Bombaim (9 km E) | 0.276333 | 6.649667 | *Schistometopum thomense* |  |  |  |  |  |  |  |
| CAS | 233460 | NA | Bombaim (9 km E) | 0.276333 | 6.649667 | *Schistometopum thomense* |  |  |  |  |  |  |  |
| CAS | 233461 | NA | Bombaim (9 km E) | 0.276333 | 6.649667 | *Schistometopum thomense* |  |  |  |  |  |  |  |
| CAS | 233462 | NA | Bombaim (9 km E) | 0.276333 | 6.649667 | *Schistometopum thomense* |  |  |  |  |  |  |  |
| CAS | 233485 | NA | Rio Maria Luisa | 0.328250 | 6.512333 | *Schistometopum thomense* |  |  |  |  |  |  |  |
| CAS | 233486 | NA | Rio Maria Luisa | 0.328250 | 6.512333 | *Schistometopum thomense* |  |  |  |  |  |  |  |
| CAS | 233487 | NA | Rio Maria Luisa | 0.328250 | 6.512333 | *Schistometopum thomense* |  |  |  |  |  |  |  |
| CAS | 233571 | NA | Lemba River bridge on coast road | 0.247333 | 6.466472 | *Schistometopum thomense* |  |  |  |  |  |  |  |
| CAS | 233572 | NA | Lemba River bridge on coast road | 0.247333 | 6.466472 | *Schistometopum thomense* |  |  |  |  |  |  |  |
| CAS | 233573 | NA | Lemba River bridge on coast road | 0.247333 | 6.466472 | *Schistometopum thomense* |  |  |  |  |  |  |  |
| CAS | 233574 | NA | Lemba River bridge on coast road | 0.247333 | 6.466472 | *Schistometopum thomense* |  |  |  |  |  |  |  |
| CAS | 233575 | NA | Lemba River bridge on coast road | 0.247333 | 6.466472 | *Schistometopum thomense* |  |  |  |  |  |  |  |
| CAS | 233576 | NA | Lemba River bridge on coast road | 0.247333 | 6.466472 | *Schistometopum thomense* |  |  |  |  |  |  |  |
| CAS | 233577 | NA | Porto Allegre | 0.035722 | 6.531389 | *Schistometopum thomense* |  |  |  |  |  |  |  |
| CAS | 233581 | NA | Porto Allegre | 0.035722 | 6.531389 | *Schistometopum thomense* |  |  |  |  |  |  |  |
| CAS | 233585 | NA | Porto Allegre | 0.035722 | 6.531389 | *Schistometopum thomense* |  |  |  |  |  |  |  |
| CAS | 233589 | NA | Porto Allegre | 0.035722 | 6.531389 | *Schistometopum thomense* |  |  |  |  |  |  |  |
| CAS | 233621 | NA | Abade | 0.255444 | 6.650472 | *Schistometopum thomense* |  |  |  |  |  |  |  |
| CAS | 233623 | NA | Abade | 0.255444 | 6.650472 | *Schistometopum thomense* |  |  |  |  |  |  |  |
| CAS | 233627 | NA | Abade | 0.255444 | 6.650472 | *Schistometopum thomense* |  |  |  |  |  |  |  |
| CAS | 233630 | NA | Abade | 0.255444 | 6.650472 | *Schistometopum thomense* |  |  |  |  |  |  |  |
| CAS | 233635 | NA | Abade | 0.255444 | 6.650472 | *Schistometopum thomense* |  |  |  |  |  |  |  |
| CAS | 233638 | NA | Abade | 0.255444 | 6.650472 | *Schistometopum thomense* |  |  |  |  |  |  |  |
| CAS | 233643 | NA | Abade | 0.255444 | 6.650472 | *Schistometopum thomense* |  |  |  |  |  |  |  |
| CAS | 233645 | NA | Abade | 0.255444 | 6.650472 | *Schistometopum thomense* |  |  |  |  |  |  |  |
| CAS | 233664 | NA | W Abade | 0.254111 | 6.644583 | *Schistometopum thomense* |  |  |  |  |  |  |  |
| CAS | 233665 | NA | W Abade | 0.254111 | 6.644583 | *Schistometopum thomense* |  |  |  |  |  |  |  |
| CAS | 233667 | NA | W Abade | 0.254111 | 6.644583 | *Schistometopum thomense* |  |  |  |  |  |  |  |
| MW | 03231 | MW03239 | Bagamoyo, Tanzania | -6.475694 | -38.822861 | *Schistometopum gregorii* | *X* |  |  |  |  |  |  |
